# Hairy cell leukemia – etiopathogenesis, diagnosis and modern therapeutic approach

**DOI:** 10.11613/BM.2024.020502

**Published:** 2024-06-15

**Authors:** Katarzyna Maćkowiak, Magdalena Jankowiak, Karolina Szewczyk-Golec, Iga Hołyńska-Iwan

**Affiliations:** 1 Department of Laboratory Diagnostic, Dr Jan Biziel University Hospital No. 2, Bydgoszcz, Poland; 2 Department of Medical Biology and Biochemistry, Faculty of Medicine, Ludwik Rydygier Collegium Medicum in Bydgoszcz, Nicolaus Copernicus University in Toruń, Bydgoszcz, Poland; 3 Department of Pathobiochemistry and Clinical Chemistry, Faculty of Pharmacy, Ludwik Rydygier Collegium Medicum in Bydgoszcz, Nicolaus Copernicus University in Toruń, Bydgoszcz, Poland

**Keywords:** BRAF V600E, hairy cell leukemia, purine analogues, cancer, molecular hematology

## Abstract

Hairy cell leukemia (HCL) represents 2% of all leukemia cases, with men aged above 55 years being the most affected. The most common symptoms of this type of leukemia include splenomegaly, monocytopenia, and neutropenia. In the basic blood count examination, leukopenia with monocytopenia and granulocytopenia, as well as aplastic anemia and/or thrombocytopenia occur. The mutation of β-rapidly accelerated fibrosarcoma (*BRAF*) proto-oncogene, which can be found in nearly 100% of patients, is an important feature of HCL. Immunophenotypic analysis of the HCL cells reveals high expression of B-lineage antigens, including CD19, CD20, and CD22. Additionally, CD11c, CD25, CD103, and CD123 belong to specific markers of HCL. Lactate dehydrogenase activity and β-2-microglobulin concentration are also important in the patient’s assessment. The differential diagnosis between HCL, hairy cell leukemia variant (HCL-V) and splenic marginal zone lymphoma (SMZL) is of first importance. Currently, the main treatment for HCL involves the use of purine analogues, excluding pregnant women, individuals with severe infections, and those with relapsing HCL.

## Graphical abstract

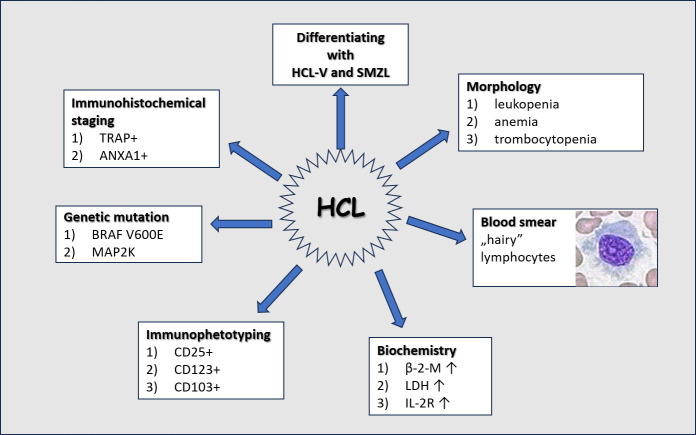

## Highlights

• A chronic lymphoproliferative disease with characteristic mature clonal B lymphocytes with hairy protrusions

• Associated with impaired activity of the B-rapidly accelerated fibrosarcoma (BRAF) protein

• Differential diagnosis of hairy cell leukemia, hairy cell leukemia variant and splenic marginal zone lymphoma allows for the selection of effective pharmacological therapy and for risk stratification

• Therapeutic approach includes purine analogues, BRAF kinase inhibitors, and anticancer immunotherapy

## Introduction

Hairy cell leukemia (HCL) is a rare chronic lymphoproliferative disorder characterized by progressive bone marrow failure due to the infiltration of mature clonal B lymphocytes with “hairy surface protrusions” (*1*-*5*). The characteristic “hairy” appearance of HCL cells is a consequence of impaired activity of the B-rapidly accelerated fibrosarcoma (*BRAF*) gene. Hair-like outgrowths on the surface of HCL cells promote their interaction with other cells or ligands. Hairy cell leukemia cells show characteristic cytomorphology, immunophenotype and molecular features (*2*, *3*). The first 26 cases of leukemic reticuloendotheliosis were described in 1958 (*5*). The BRAF V600E mutation occurs in virtually 100% of cases of classic HCL and is considered a disease-defining event (*2*, *4*). This mutation is not present in a HCL variant (*4*). Hairy cell leukemia variant (HCL-V) is a newly discovered separate disease entity that differs from the classic form in terms of morphology, genetics and immunophenotype (*4*).

Currently, HCL accounts for 2% to 3% of all leukemias (*3*, *6*). The annual incidence of HCL ranges from 2.9 to 4.7 cases *per* million people worldwide (*7*, *8*). Men are affected more often than women (4:1), and the median age at diagnosis is 55 years (*8*-*10*). Caucasians are affected more often than other ethnic groups (*3*, *10*). Patients usually present clear clinical features (*1*, *7*, *9*, *10*). Typical symptoms include splenomegaly, leukopenia with monocytopenia in the blood count, and a characteristic microscopic image of lymphoid cells with “disheveled” cytoplasm (*1*, *5*, *6*, *9*-*12*).

This review provides an overview of the recommended and used diagnostic methods for HCL and a summary of the latest therapeutic procedures. Due to the fact that in recent years there have been significant changes in the diagnostic and therapeutic approach, especially related to the differentiation of HCL from HCL-V and splenic marginal zone lymphoma (SMZL), this issue was given special attention in this review.

## Etiopathogenesis of hairy cell leukemia

**Figure 1:**
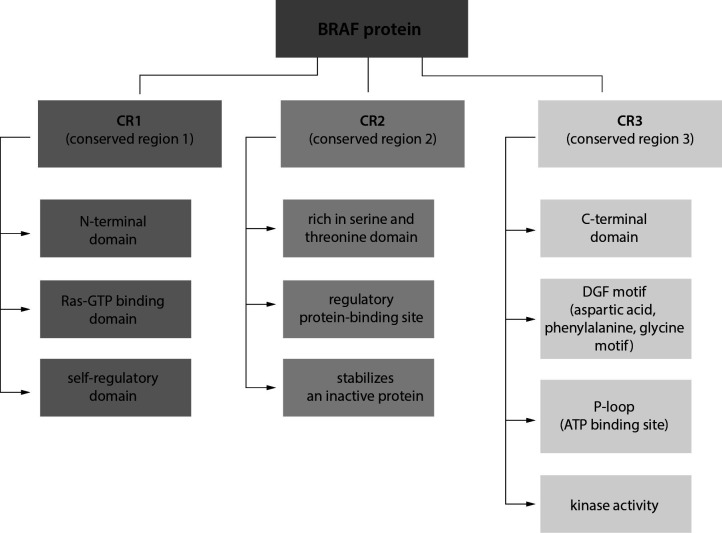
The structure and function of domains of B-rapidly accelerated fibrosarcoma (BRAF) protein.

**Figure 2:**
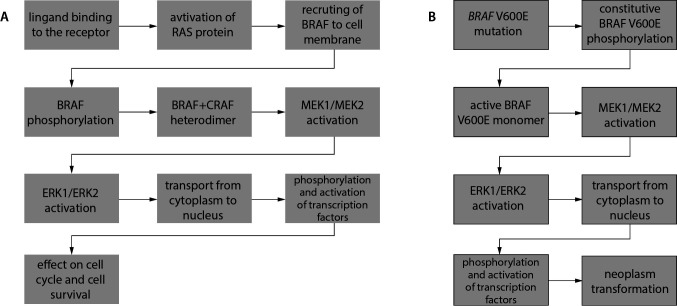
The model of neoplastic transformation of hairy cell leukemia: A – the physiological regulatory pathway initiated by the phosphorylation of the B-rapidly accelerated fibrosarcoma (BRAF) protein, B – the pathological activation of the pathway initiated by the V600E-mutated BRAF protein. CRAF – C-rapidly accelerated fibrosarcoma. ERK1/2 – extracellular signal regulated kinase 1/2. MEK1/2 – mitogen activated protein kinase 1/2. RAS – rat sarcoma virus.

As a result of genome sequencing, it has been found that the mutation in the *BRAF* gene occurs in a significant population of HCL patients, ranging 70-100% of the patients (*3*, *6*, *11*, *12*). The BRAF protein is encoded by a proto-oncogene located on the chromosome 7q24 and belongs to the RAF protein-serine/threonine kinase family (EC. 2.7.11.1). The BRAF protein is composed of three domains, namely conserved region 1 (CR1), CR2, and CR3 (Figure 1) (*13*). Conserved region 1 is an N-terminal self-regulatory domain. Conserved region 2 contains a regulatory protein-binding site that stabilizes an inactive state of BRAF. Conserved region 3 is a C-terminal domain, which contains an activation segment and an adenosine 5’-triphosphate (ATP)-binding site, so-called a phosphate-binding loop (P-loop). In an inactive conformation, an access to the catalytic site of BRAF is blocked due to hydrophobic interactions (*11*). The activation of BRAF occurs through the phosphorylation of the activation segment, the process mediated by the RAS protein (*14*). Regulation of BRAF activity in physiological and pathological conditions is summarized in Table 1 and Figure 2. It is noteworthy that the serine residue at position 445 of BRAF is constitutively phosphorylated, unlike the homologous serine residue at position 338 of C-rapidly accelerated fibrosarcoma (CRAF) protein (*11*-*14*). This feature explains why a single mutation at codon 600 (V600E) is sufficient for continuous BRAF activation and why BRAF is the most frequently mutated RAF protein in human cancers (*11*, *15*). The mentioned mutation in the *BRAF* gene contributes to the constitutive activation of RAS-RAF-MEK-ERK signaling pathway, which is the mitogen activated kinase system involved in the cell proliferation and tumor progression (*1*, *11*, *12*, *16*). It has been shown that the activity of the BRAF V600E-mutated protein is 480 times greater than the basic one and that the BRAF protein has a 5-fold greater ability to activate kinases compared to an unmutated form (*16*).

**Table 1:** Regulation of B-rapidly accelerated fibrosarcoma (BRAF) protein activity in physiology and pathology

**Condition**	**Mechanism**	**Consequences**
Inactive BRAF	Hydrophobic interactions of DFG motif and P-loop	Access to catalytic site blocked
Active BRAF	Phosphorylation modifies hydrophobic interactions of DFG motif with P-loop	Catalytic site open
Hairy cell leukemia (V600E mutation of BRAF gene, exon 15, 1799 position)	Hydrophobic interactions are lacking due to the replacement of valine with glutamine	Catalytic site constantly open
DFG – aspartic acid, phenylalanine and glycine motif in conserved region 3 of BRAF protein. P-loop – adenosine 5’-triphosphate binding loop in conserved region 3 of BRAF protein.		

The first stage of the pathway involves the formation of an active heterodimer composed of the phosphorylated BRAF and CRAF (*11*-*13*). The next step includes the phosphorylation and activation of mitogen activated protein kinase 1 (MEK1) and 2 (MEK2). Activated MEK1/MEK2 regulates extracellular signals by the phosphorylation of extracellular signal regulated kinase 1 (ERK1) and 2 (ERK2), which can translocate from the cytoplasm to the cell nucleus. In the nucleus, they can phosphorylate the transcription factor activating proteins 2 (TFAP2) and the nuclear factor of active T cells (NFAT), affecting the transcription of genes that promote the cell cycle progression and cell survival (*11*). It should be added that fibroblast growth factor (FGF) and cyclin D1 also play important roles in this pathway. Fibroblast growth factor is a proto-oncogene strongly involved in the growth of hairy cells, because HCL cells not only participate in its secretion, but they also have the fibroblast growth factor receptor 1 (FGFR1). Activation of FGFR1 triggers the ERK signal leading to the cell cycle progression and transformation of fibroblasts (*1*, *11*, *12*, *17*). It has been shown in *in vivo* experiments that the activation of the RAS-RAF-MEK-ERK pathway in HCL patients is mediated by the expression of cyclin D1 and the ERK protein in hairy cells of the bone marrow (*11*, *17*). Both the ERK protein and cyclin D1 affect the cell proliferation; thus, their improper expression may lead to the neoplastic transformation (*1*, *17*).

The key role of the V600E mutation of the *BRAF* gene in the etiopathogenesis of HCL is confirmed by the fact that this mutation can be detected in anatomical sites closely related to HCL, such as lymph nodes (*1*, *11*, *12*, *17*). It has been observed that the V600E mutation is very stable over time and can be detected in disease recurrence, even several years after initial diagnosis (*9*). Recurrent mutations in the genes coding for the enhancer of Zeste 2 (EZH2), AT-rich interaction domain 1A (ARID1A), and cyclin-dependent kinase inhibitor 1B (CDKN1B) have also been identified in patients with HCL resistant to purine analogues (*11*). Cyclin-dependent kinase inhibitor 1B is involved in the control of the cell cycle and might play an important role in the pathogenesis of HCL (*1*, *11*, *12*, *17*). Mutations of CDKN1B have been detected in 16% of cases (*1*, *10*).

### Cell effects of the BRAF mutation

The conducted studies explaining the origin of the characteristic “cytoplasmic hairs” of HCL cells have confirmed that the hairy cell is a consequence of dysregulated BRAF activity (*11*). Acquiring such a unique feature allows the cells to increase their surface area, which favors their interaction with other cells or ligands in the extracellular matrix. *In vitro* exposure of primary HCL cells to BRAF or MEK inhibitors has been found to convert their morphology from hairy to smooth (*11*, *18*, *19*). An important role is played by F-actin found in the polarized form of fibrillar actin, which is present at the periphery of the cell to maintain the structure of the protrusion (*19*). In normal B lymphocytes, F-actin is located mainly in the central part of the cells. Hairy cell leukemia cells overexpress selected components of the cytoskeleton, including F-actin and intracellular phosphoproteins involved in the active reorganization of the cytoskeleton (*1*, *11*, *12*, *19*).

### Hairy cell leukemia and the *Coxiella burnetii* infection

The influence of *Coxiella burnetii* bacteria on the transformation of human B lymphocytes into hairy cells has been highlighted in some studies (*19*, *20*). In 1993, Lee *et al.* observed that *C. burnetii* induced the transformation of lymphocytes into hairy cells, which did not differ morphologically from the cells described in the course of HCL (*19*). Further development of molecular biology techniques made it possible to prove the involvement of *C. burnetii* in inducing the formation of hairy cells and inhibiting apoptosis (*19*, *20*). The pathogen induces the reorganization of the cell cytoskeleton (*19*). Studies carried out in France and Germany confirmed a significant increase in the risk of developing non-Hodgkin lymphomas (NHL) among patients who had the *C. burnetii* infection compared to healthy people (*20*). The reservoir of the pathogen consists of infected domestic cattle and animal derivatives, including milk, hair, feces, and placenta (*19*, *21*). Therefore, there is an increased risk of infection for people who work on farms or come into a direct contact with contaminated products. Veterinarians, employees of fur and butchery factories are at risk of infection transmission (*19*, *21*). The risk of transmission by ticks, which once infected become carriers for life, is also significant (*21*). It is worth mentioning that *C. burnetii* can survive in tick feces for up to 6 years (*21*).

### Influence of the bone marrow microenvironment on hairy cells

Hairy cell leukemia cells are characterized by high expression of receptors for molecules such as C-X-C chemokine receptor type 4 (CXCR4), very late antigen-4 (VLA-4), adhesion molecules including integrins and CD44, B cell antigen receptor (BCR), and CD40 antigen (*6*, *22*, *23*). Under physiological conditions, chemokines play an important role in the processes of migration, adhesion and retention of progenitor cells in the bone marrow (*22*). Cancerous B cells can use these molecules to gain access to protective niches in the bone marrow (*1*). Contact of bone marrow stromal cells (BMSC) with malignant B lymphocytes may induce drug resistance, increase the risk of disease recurrence and, consequently, the occurrence of minimal residual disease (MRD) (*23*). The use of standard pharmacotherapy allows the elimination of most cancer cells; however, “residual” hairy cells can hide in protective niches and still receive signals promoting their survival and proliferation. The interaction between HCL cells and elements of the bone marrow microenvironment can lead to the activation of the mitogen-activated protein kinase (MAPK) pathway and the nuclear factor kappa B (NF-κB) pathway (*1*, *22*). One study has highlighted the usefulness of CXCR4 chemokine antagonists in the treatment of HCL, indicating that their use may increase hairy cell exposure to drugs (*23*). However, other normal progenitor cells are also exposed to the cytotoxic effects of the drug, which is problematic. It would be advisable to determine whether progenitor cells have the same mobilization threshold as normal stem cells. This aspect is a subject to further research (*23*).

Bone marrow stromal cells continuously secrete numerous chemokines and express ligands for various adhesion molecules, leading to the activation of signaling pathways involved in the survival and growth of HCL cells, including phosphatidyl inositol 3-kinase (PI3K-AKT), V-akt murine thymoma viral oncogene homolog 1 (AKT1), protein kinase C (PKC), and BCR signaling pathways (*1*, *3*, *17*). Culture of HCL cells with BMSC has been found to have the ability to evade apoptosis induced by interferon alpha (IFN-α) or BRAF inhibitors (*1*, *12*, *18*). It has been observed that hematopoietic stem cells (HSCs) and B lymphocyte progenitors taken from HCL patients belong to BRAF mutation carriers (*11*, *18*). After the implantation of mutant stem cells into immunodeficient mice, HCL cells were able to self-renew (*11*). It is significant that none of the animals subjected to the experiment developed the full HCL phenotype. The conducted experiment showed that the etiopathogenesis of HCL is complex and results from overlapping of various factors (*1*, *11*). The multifactorial pathogenesis of HCL is primarily associated with the overlap of genetic mutations, in particular in the *BRAF* gene, with cytoskeleton reorganization disorders and *C. burnetii* infection, which can most likely lead to the development of a full-blown disease. The isolated *BRAF* genetic mutation most likely induces the cell senescence and apoptosis, and not the progression and transformation of HCL (*1*).

## Symptoms of hairy cell leukemia

The main clinical symptoms faced by HCL patients include fatigue (80%) and abdominal pain accompanied by loss of appetite, which is a consequence of splenomegaly, characteristic of 80-90% of the patients (*3*, *10*, *24*, *25*). A significant percentage of patients struggle with recurrent infections, often with severe course, fever (15-40%), and bleeding, which is the result of thrombocytopenia (*3*, *10*, *24*, *25*). Some patients (10-12%) develop skin lesions in the form of papules, nodules, and painful erythematous lesions, occasionally with central ulceration, usually resulting from autoimmune or infectious causes (*7*, *9*, *10*, *26*). A direct infiltration of the skin by HCL cells is an unusual phenomenon (*5*). In rare cases, dermatoses are the first manifestation of HCL (*7*). One of the accompanying symptoms is permanent bone pain resulting from osteolytic changes (*3*, *9*, *26*). Destructive bone changes in patients with HCL were first described in 1977 (*26*). The report concerned three patients with bone pain developing within 2-3 years of initial diagnosis. Lytic bone changes most often affect the axial skeleton. They usually appear in bones of the skull, neck and femoral neck and can affect about 3% of the patients (*5*, *9*, *10*, *26*).

## Diagnostics of hairy cell leukemia

The diagnosis of HCL is based on a complex blood count assessment with a microscopic smear, and the examination of the bone marrow, considering its morphological, immunohistochemical, and immunophenotypic features (*1*, *4*, *10*, *18*, *27*). To assess the severity of the disease, imaging tests are recommended, including computed tomography and ultrasound (*10*, *27*).

In the basic blood count examination, approximately 80% of patients have leukopenia with monocytopenia and granulocytopenia, whereas one third of patients suffer from aplastic anemia and/or thrombocytopenia (*9*, *10*, *28*).

**Figure 3:**
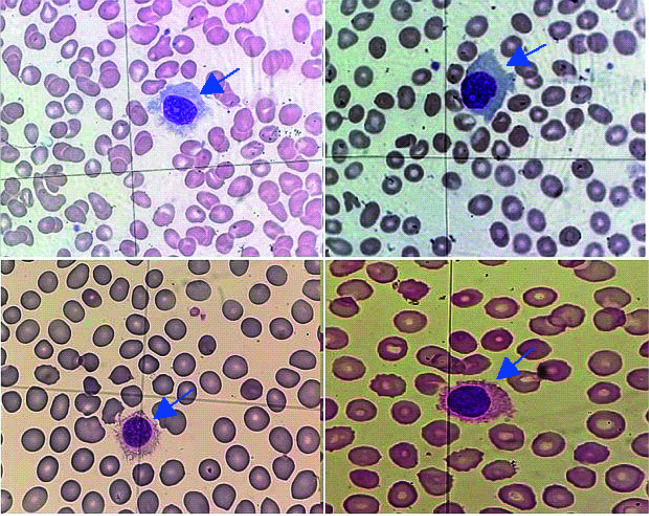
Peripheral blood smears of hairy cell leukemia patients showing lymphoid cells with hairy projections (blue arrows). May-Grünwald-Giemsa staining x100 (photos from the authors’ own collection).

In the microscopic image, characteristic lymphoid cells, so-called hairy cells, are observed (Figure 3). Hairy cells have a diameter of 10 to 25 µm, a round, oval, or bean-shaped cell nucleus with sparse chromatin, and abundant, pale blue and jagged cytoplasm with shaggy projections. Occasionally, fine granules may be found (*9*, *10*, *18*, *24*). Due to their size, hairy cells are often misidentified by the hematology analyzer as monocytes; therefore, an important part of diagnostics is the microscopic evaluation of preparations by qualified personnel and, based on this, the determination of the actual leukocyte percentage formula (*9*, *10*).

### Biochemical tests in hairy cell leukemia

Among the biochemical parameters useful in assessing the course of HCL, β-2-microglobulin (β-2-M) and lactate dehydrogenase (LD, E.C.1.1.1.27) play an important role (*9*). Both parameters show an increase in the progression of lymphoproliferative diseases. β-2-microglobulin correlates positively with tumor mass; its increased concentrations may be associated with a worsening of the patient’s condition and higher risk of recurrence. For LD, the optimal cut-off point has been estimated at 200.5 IU with a diagnostic sensitivity of 73% and specificity of 61% (*29*, *30*). Lactate dehydrogenase activity exceeding 200.5 IU is associated with a higher risk of relapses and shortened overall survival (*29*, *30*).

Considering biochemical tests, it is also recommended to determine parameters evaluating the liver function, including activities of aspartate aminotransferase (AST, EC.2.6.1.1) and alanine aminotransferase (ALT, EC.2.6.1.2), the concentration of bilirubin, as well as renal markers, including the concentration of creatinine and urea, and the estimation of glomerular filtration rate (eGFR) (*4*, *9*, *10*, *31*).

More than 90% of HCL patients have bone marrow involvement, as HCL cells tend to infiltrate tissues rich in hyaluronate (*26*, *27*). The bone marrow, initially rich in cells, may become hypoplastic and then aplastic as the disease progresses (*4*, *9*, *10*). This process is caused by the increased level of cytokines released by hairy cells, including transforming growth factor (TGF-β1) and tumor necrosis factor α (TNF-α) (*1*, *22*, *23*). The mentioned cytokines stimulate fibroblasts to synthesize reticulin fibers, causing gradual myelofibrosis and, consequently, pancytopenia in the peripheral blood smear. Due to reactive myelofibrosis, trephine biopsy is recommended for diagnostic purposes (*3*, *9*, *18*). Patients with pancytopenia are at risk of severe opportunistic infections, therefore, they should have monitored inflammatory parameters such as C-reactive protein (CRP), procalcitonin (PCT) and interleukin 6 (IL-6). Currently, research is underway on the usefulness of determining the soluble form of the α-chain of the interleukin 2 receptor (IL-2R). It is postulated that the level of IL-2R correlates with the tumor mass (*32*). A comprehensive assessment of the patient condition supplemented with biochemical and immunochemical tests allows for monitoring the course of the disease and assessing the risk of complications and recurrence.

### Immunohistochemical staining in hairy cell leukemia

Immunohistochemical staining of bone marrow aspirates is useful to confirm the diagnosis of HCL. Basic tests include the assessment of the phosphatase reaction. In HCL cells, acid phosphatase 5 isoenzyme (ACP5, EC.3.2.3.2), which is insensitive to tartrate, predominates. Positive tartrate-resistant acid phosphatase (TRAP) reaction is used in the diagnostic process. Double labeling for both paired box 5 (Pax5) in the cell nucleus and annexin A1 (ANXA1) in the cytoplasm with the use of monoclonal antibodies against ANXA1 epitopes and fixation-resistant TRAP or CD103, which are highly specific for HCL, is the preferred procedure (*1*, *10*, *33*-*35*). It should be noted that ANXA1 is an important diagnostic marker, but it is not useful for monitoring residual disease after therapy (*11*, *28*, *33*). Detection of small amounts of ANXA1 in positive HCL cells is hampered by the presence of a large population of myeloid elements, macrophages and T cells, which also express this protein (*34*). Bone marrow aspirates can also be subjected to immunohistochemical staining using a specific monoclonal antibody against the *BRAF* V600E (VE1) or CD72 mutations (*10*, *35*).

In exceedingly rare cases, leukemia cells do not infiltrate the bone marrow, but infiltrate the spleen in isolation. The splenic infiltrates are confined to the red pulp, in which the blood-filled pseudosinuses are lined with hairy cells. It should be noted that splenomegaly is a consequence of red pulp hypertrophy, while white pulp is atrophied (*1*, *9*).

### Immunophenotyping in hairy cell leukemia

Immunophenotyping using flow cytometry is a sensitive and specific method in the HCL diagnostics (*10*, *33*). Hairy cell leukemia cells highly express B-lineage antigens, including CD19, CD20, and CD22. Additionally, specific markers for this disease entity include CD11c, CD25, CD103, and CD123 (*9*, *10*, *28*, *36*). In the differentiation process, one point is assigned for each individually confirmed antigen. The total score is 3-4 points for HCL, whereas, for HCL-V and SMZL, the sum is 0-2 points (*6*, *18*, *37*). A score above 3 points is strongly positively correlated with the diagnosis of HCL, covering 98% of patients (*3*, *37*). Previous research results have shown that the expression of the CD200 antigen is highly specific for HCL (*3*, *37*). It should be noticed that CD38 is an unfavorable marker of the classic form of the disease, because its expression may be associated with a higher risk of recurrence (*10*, *18*, *35*). In the course of HCL, the number of peripheral T lymphocytes is usually reduced, with an inverse CD4 to CD8 ratio and reduced expression of CD28, probably contributing to the development of secondary immunodeficiencies (*1*).

The detection of a mutation in the *BRAF* gene is of key importance in confirming the diagnosis of HCL. This mutation affects as many as 70-100% of the patients, whereas mutations in the cyclin dependent kinase (CDKN) or transcription factor 2 (KLF2) genes can be found in about 16% of the patients (*1*, *11*). *BRAF* genetic changes are not specific to HCL, because they can be detected in other disease entities, *e.g.,* melanoma, cancer of the lung, intestine, thyroid gland, and, much less frequently, in hematological hyperplasias (*6*, *10*, *18*). Additionally, somatic hypermutation of the variable regions of genes coding for immunoglobulin heavy chains (IGHV) is of significant importance, occurring in approximately 90% of HCL patients (*1*-*3*).

### Differential diagnosis of hairy cell leukemia

The differentiation of the classic form of HCL from HCL-V and SMZL plays an important role, allowing the selection of an appropriate therapeutic path and assessment of the risk of recurrence.

Hairy cell leukemia variant accounts for 0.4% of all leukemias (*26*, *31*). It was first described in 1980 by Cawley *et al.* (*34*). The median age at diagnosis of HCL-V, equal to 71 years, is higher than that of HCL (*31*). The course of the disease is more aggressive compared to HCL, with worse prognosis and shorter overall survival (*6*, *10*, *35*). In HCL-V, leukocytosis may reach very high values in the range of 20-40 x10^9^/L (*37*-*39*). In the microscopic image, lymphoid cells with features intermediate between a prolymphocyte and a hairy cell can be found (*5*). The feature that distinguishes these cells from classical HCL cells includes the presence of a nucleolus, which is pronounced in 62% of cases and smaller and inconspicuous in 38% of cases (*29*, *38*). The bone marrow is rich-cell, less often hypoplastic, with a much lower degree of infiltration compared to the classic form of the disease (*29*, *39*). Both classic and variant HCL are characterized by splenomegaly with red pulp infiltration (*1*, *5*, *38*). Immunohistochemical, cytometric molecular research using the polymerase chain reaction (PCR) helps differentiate HCL from HCL-V (Table 2) (*11*, *18*, *28*, *38*, *39*). In HCL-V, lack of *BRAF* mutations is observed, which is also associated with the nodal form of the disease and a weaker response to the treatment with purine analogues (*10*, *35*, *38*). Additionally, it is recommended to evaluate mutations of the tumor protein P53 (*TP53*) gene, which concerns about 30% of the patients and is associated with resistance to standard therapy (*3*, *11*, *18*).

**Table 2:** Comparison of features of hairy cell leukemia (HCL), variant hairy cell leukemia (HCL-V) and splenic marginal zone lymphoma (SMZL)

	**HCL**	**HCL-V**	**SMZL**
Blood count	Leukopenia, monocytopenia	High leukocytosis, without monocytopenia	Leukocytosis, without monocytopenia
Microscopic smear of peripheral blood	1. Nucleus – round, oval or bean-shaped with less condensed chromatin. 2. Nucleolus - not present. 3. Cytoplasm - pale blue, characteristically jagged (ruffled).	1. Nucleus - round or oval, with less condensed chromatin. 2. Nucleolus - present. 3. Basophilic cytoplasm, jagged cytoplasm less pronounced and more delicate.	1. Nucleus - irregular with a stronger degree of chromatin condensation 2. Nucleolus - poor visible. 3. Cytoplasm - short cytoplasmic projections polarly arranged, visible in 1/3 of cases.
Immunophenotyping	CD25+ CD123+ CD103+	CD25– CD123+/– CD103+	CD123– CD103–
Genetic mutations (% of patients)	BRAF (100%) MAP2K (0-22%)	BRAF (0%) CCND3 (13%) MAP2K (38-40%)	BRAF (0%) KLF2 (20-30%) MAP2K (0%)
Immunohistochemical staging (% of patients)	TRAP+ (95%) ANXA1+ (100%)	TRAP+ (38%) ANXA1– (0%)	–
Percentage of the bone marrow infiltration (% of patients)	75-100% (73%) 25-75% (22%) < 25% (5%)	75-100% (21%) 25-75% (50%) < 25% (29%)	Not present
Spleen infiltration	Red pulp	Red pulp	White pulp
BRAF - B-rapidly accelerated fibrosarcoma. TRAP - tartrate-resistant acid phosphatase. ANXA1 - annexin A1. CD - cluster of differentiation. MAP2K - mitogen activated protein 2 kinase. KLF2 – KLF transcription factor 2. CCND3 – cyclin D3.			

**Figure 4:**
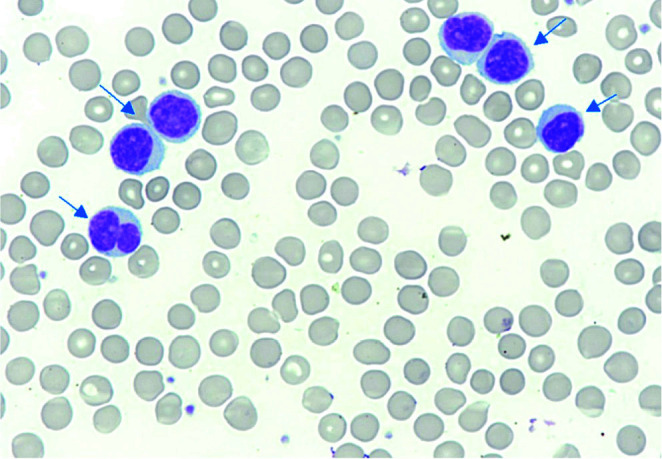
Peripheral blood smear of a splenic marginal zone lymphoma patient showing atypical lymphoid cells (blue arrows). May-Grünwald-Giemsa staining x100 (photo from the authors’ own collection).

Differentiation between HCL and SMZL is also very important. Splenic marginal zone lymphoma accounts for approximately 1% of all lymphocytic neoplasms (*40*, *41*). This lymphoma is characterized by significant splenomegaly and increased lymphocytosis reaching 10-30 x10^9^/L (*40*). In SMZL, leukemic cells infiltrate the white pulp of the spleen. In the microscopic image, small lymphoid cells with short cytoplasmic protrusions can be observed (Figure 4) (*40*, *41*). Phenotypically, cancer cells express antigens typical of the B lineage and surface immunoglobulins IgD or IgM, as well as CD29 and CD79a particles (*40*, *42*). However, there is no expression of antigens CD103 and CD123 and nuclear cyclin D1 (*41*). The PCR method can detect a mutation in the *KLF2* gene and no changes in the *BRAF* and *MAP2K* genes (*11*, *12*).

The distinction between HCL, HCL-V and SMZL plays a priority role, allowing for the selection of appropriate, effective pharmacological therapy and for risk stratification, *i.e.,* the assessment of prognosis, risk of recurrence, and a chance of complete remission (*6*, *38*, *39*, *41*, *43*).

## Therapeutic approach in hairy cell leukemia

Initiation of HCL treatment depends on the stage of the disease. This type of leukemia is highly treatable, but pharmacotherapy is rarely implemented (*3*, *43*). The decision to treat depends on the stage of the disease. In order to assess the stage of the disease, several aspects are taken into account, such as splenomegaly, tumor mass, degree of bone marrow infiltration, increased risk of infectious complications, and changes in blood counts, including platelets < 100 x10^9^/L, hemoglobin < 10 g/dL, and neutropenia < 1 x10^9^/L (*4*, *42*, *44*).

If a patient is asymptomatic and the blood count remains normal, treatment may not be necessary. However, it is advisable to monitor untreated patients every 3-6 months, with particular attention to medical history, physical examinations and blood counts (*28*, *31*).

Chemotherapy with cladribine (2-chloro-2’-deoxyadenosine) and pentostatin (deoxycoformicin) is the most commonly used option for the treatment of HCL. Cladribine and pentostatin belong to purine antimetabolites that mimic the nucleotide adenosine and inhibit the enzyme adenosine deaminase (EC.3.5.4.4) (*18*, *41*, *44*, *45*). This therapy is used especially in young patients (*25*, *31*, *44*). Usually, after 8-9 cycles of chemotherapy, the normalization of blood count parameters can be observed. However, a bone marrow biopsy is needed to confirm complete response (CR) (*4*, *33*). For more than 30 years, cladribine and pentostatin have remained the first-line drugs in HCL patients with an overall response rate (ORR) > 90%, CR > 75%, and long-term progression-free survival (PFS) longer than 20 years for people in deep remission (*35*, *45*). Recently, it was determined that the most effective therapy in previously treated patients who had their first relapse includes cladribine together with vemurafenib, a small-molecule sulfonamide derivative (*4*, *33*). Vemurafenib, a targeted cancer drug, is a highly selective BRAF serine/threonine kinase inhibitor used to treat advanced melanoma and other skin cancers. The combination cladribine/vemurafenib is used in treatment-resistant cases (*27*, *46*). Additionally, the recombinant IFN-α can be used in the HCL treatment, especially in pregnant women, because it is safe for the fetal development and does not cause complications during childbirth. The IFN-α treatment is also beneficial for patients with severe neutropenia, purine analogue ineligibility, and relapsed/refractory HCL. However, its use is limited due to its low CR- (*1*, *18*, *25*, *31*, *42*, *47*).

The subsequent side effect of the use of purine antimetabolites is myelo- and immunosuppression, which translates into longer reconstruction of hematopoiesis, periodically deepened cytopenia and greater susceptibility to infections (*38*). However, an indisputable advantage of purine analogues is the possibility of their administration during an active infection and adjusting the dose during treatment (*46*, *47*). Immunosuppression acquired by the use of cladribine can result in long-term neutropenia and impaired T cell function. Therefore, the Hairy Cell Leukemia Foundation proposed the use of rituximab as an alternative to BRAF inhibitors or in combination with BRAF inhibitors (*37*). Rituximab is an example of an immunotherapeutic drug with an anticancer and immunosuppressive effect. It is a human-mouse chimeric anti-CD20 monoclonal antibody produced by genetic engineering (*47*). Rituximab binds specifically to the transmembrane cell differentiation antigen CD20, which is a non-glycosylated phosphoprotein found on both pre-B lymphocytes and mature B lymphocytes. Importantly, better results have been observed after combining rituximab with alemtuzumab may be another treatment option. Alemtuzumab is a humanized IgG1 kappa antibody containing the variable and constant regions of a human antibody, as well as the complementarity-determining regions of a rat monoclonal antibody directed against the cell membrane glycoprotein CD52 (*46*). Initially known as Campath, and later MabCampath, it has been used to treat patients with B-cell chronic lymphocytic leukemia. This drug can also cause side effects, such as an increased risk of infection in patients (*46*).

Splenectomy is not considered a first-line treatment for HCL (*46*, *47*). It is used to treat the complications of splenomegaly that can occur in HCL. In both first-line and later treatment, splenectomy could correct cytopenia and alleviate the course of the disease (*40*, *46*). Radiotherapy may be an alternative treatment option helpful in patients with hypersplenism and splenic pain who have lympho- or myeloproliferative disorders (*46*). Approximately 85-90% of patients treated with targeted radiotherapy achieve the resolution of symptomatic splenomegaly. Therefore, radiotherapy may be a useful alternative for old patients or those who are not eligible for surgery. Moreover, it is a solution for pregnant women with HCL progression and for patients resistant to nucleoside analogues or IFN-α (*33*, *34*). Nevertheless, it should also be considered that radiotherapy may lead to long-term post-radiation complications, such as anemia, ulceration or extensive tissue fibrosis (*31*, *33*).

Allogenic transplantation is an alternative treatment option for HCL in young patients, as well as in severely overtreated patients, those with multiple relapses or those resistant to purine analogues and rituximab (*9*, *31*, *41*).

The BRAF inhibitors are considered a novelty in therapy. As kinase inhibitors, they act by blocking the activity of the mutant BRAF protein (*11*). Two most commonly used BRAF inhibitors in the treatment of HCL include vemurafenib and dabrafenib. These drugs can destroy leukemic cells with a unique molecular and morphological identity, leading to their apoptosis (*18*). Clinical studies have shown high effectiveness of BRAF inhibitors in the treatment of HCL with the *BRAF* V600E mutation (*11*, *46*). Vemurafenib has side effects, such as joint pain and inflammation, rash, photosensitivity, and increased activity of pancreatic enzymes, namely amylase (EC.3.2.1.1) and lipase (EC.3.1.1.3) (*40*).

**Figure 5:**
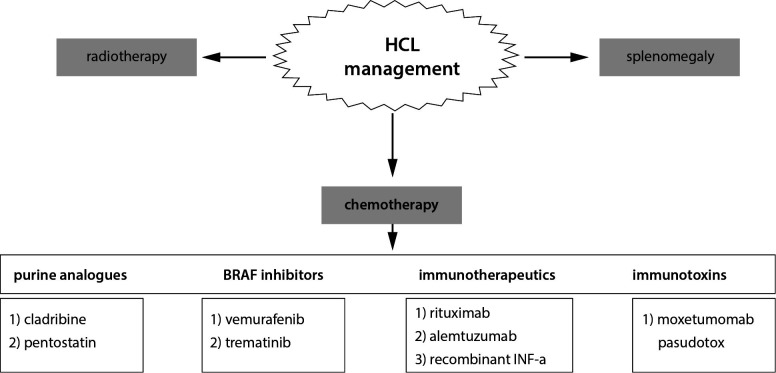
Current therapeutic options in the treatment of hairy cell leukemia (HCL). BRAF – B-rapidly accelerated fibrosarcoma protein. IFN-α – interferon-alpha.

The combination of a BRAF inhibitor with a MEK inhibitor, *e.g.*, trematinib, for the treatment of HCL-V and HCL patients with mutations in the *VH4-34* gene may be an appropriate strategy for the future (*41*, *46*). Moxetumomab pasudotox is another innovating drug, which is an immunotoxin created by the fusion of bacterial toxin with a monoclonal antibody directed against a specific target on cell surface. The drug works by binding to CD22 on cancer cells and delivering *Pseudomonas* exotoxin A to the cells, causing them to be destroyed (*43*, *46*). The mentioned drugs are currently unavailable in Europe. Current therapeutic options for the treatment of HCL are summarized in Figure 5.

## Conclusion

Hairy cell leukemia is undeniably a significant diagnostic problem, because its symptoms can mimic other lymphoproliferative disorders. In the diagnosis of HCL, it is important to use a number of parameters with confirmed significance, using traditional and modern methods such as blood smear, blood count, immunohistochemical staging, immunophenotyping, biochemical tests, and analysis of genetic mutations. The results of the mentioned diagnostic tests are crucial for proper diagnosis, differentiating with HCL-V and SMZL, selection of effective therapy and assessment of patients. The use of purine analogues makes HCL a disease with a high cure rate. However, since the BRAF proto-oncogene mutation can be found in almost 100% of HCL patients, targeted cancer drugs belonging to selective BRAF kinase inhibitors are considered in the treatment of patients with relapses. Advanced immunotherapeutics can also be used, especially combined with purine analogues. Undoubtedly, further research on the molecular etiopathology of HCL is needed to better understand the disease, improve diagnosis and personalize the patient’s treatment.

## Data Availability

No data was generated during this study, so data sharing statement is not applicable to this article.
